# Predicting lung cancer survival prognosis based on the conditional survival bayesian network

**DOI:** 10.1186/s12874-023-02043-y

**Published:** 2024-01-22

**Authors:** Lu Zhong, Fan Yang, Shanshan Sun, Lijie Wang, Hong Yu, Xiushan Nie, Ailing Liu, Ning Xu, Lanfang Zhang, Mingjuan Zhang, Yue Qi, Huaijun Ji, Guiyuan Liu, Huan Zhao, Yinan Jiang, Jingyi Li, Chengcun Song, Xin Yu, Liu Yang, Jinchao Yu, Hu Feng, Xiaolei Guo, Fujun Yang, Fuzhong Xue

**Affiliations:** 1 Department of Epidemiology and Health Statistics, School of Public Health, Cheeloo College of Medicine, Shandong University, Jinan, China; 2 Hainan Center for Disease Control and Prevention, Institute for Prevention and Control of Tropical Diseases and Chronic Noninfectious Diseases, Haikou, Hainan China; 3 Institute for Medical Dataology, Shandong University, Jinan, China; 4 Department of Oncology, Weihai Municipal Hospital, Cheeloo College of Medicine, Shandong University, Weihai, China; 5 Chongqing Key Laboratory of Computational Intelligence, Chongqing University of Posts and Telecommunications, Chongqing, China; 6 School of Computer Science and Technology, Shandong Jianzhu University, Jinan, China; 7 Department of Pulmonary and Critical Care Medicine, Weihai Municipal Hospital, Cheeloo College of Medicine, Shandong University, Weihai, China; 8 Department of Thoracic Surgery, Weihai Municipal Hospital, Cheeloo College of Medicine, Shandong University, Weihai, China; 9 Department of Radiology, Weihai Municipal Hospital, Cheeloo College of Medicine, Shandong University, Weihai, China; 10 The Second School of Clinical Medicine of Binzhou Medical University, Yantai, China; 11 The Department for Chronic and Non-communicable Disease Control and Prevention, Shandong Center for Disease Control and Prevention, Jinan, China

**Keywords:** Lung cancer, Prediction model, Missing data imputation, Bayesian Network, Cox proportional hazards model

## Abstract

Lung cancer is a leading cause of cancer deaths and imposes an enormous economic burden on patients. It is important to develop an accurate risk assessment model to determine the appropriate treatment for patients after an initial lung cancer diagnosis. The Cox proportional hazards model is mainly employed in survival analysis. However, real-world medical data are usually incomplete, posing a great challenge to the application of this model. Commonly used imputation methods cannot achieve sufficient accuracy when data are missing, so we investigated novel methods for the development of clinical prediction models. In this article, we present a novel model for survival prediction in missing scenarios. We collected data from 5,240 patients diagnosed with lung cancer at the Weihai Municipal Hospital, China. Then, we applied a joint model that combined a BN and a Cox model to predict mortality risk in individual patients with lung cancer. The established prognostic model achieved good predictive performance in discrimination and calibration. We showed that combining the BN with the Cox proportional hazards model is highly beneficial and provides a more efficient tool for risk prediction.

**Supplementary Information:**

The online version contains supplementary material available at 10.1186/s12874-023-02043-y.

## Introduction

Lung cancer remains a significant public health issue and has become the most common cancer worldwide [1]. According to Global Cancer Statistics 2020 from GLOBOCAN, lung cancer accounts for 17.9% of all cancers in China [2]. Regarding mortality, lung cancer accounts for 19.4% of cancer deaths in China [3]. Given the high incidence and mortality rates, quantifying the risk of lung cancer deaths is crucial. Personalized prognostic models play a significant role in clinical decision-making, especially in cancer research, by exploring the relationship between predictive factors and outcome risks. Currently, many individuals undergo annual physical examinations, and electronic health records (EHRs) have collected vast amounts of data, which are essential for researching lung cancer prognostication. We collected EHR data from lung cancer patients diagnosed at Weihai Municipal Hospital in China to predict the overall survival of lung cancer patients.

The previous study focused on establishing a survival prediction model for lung cancer using regression methods such as Cox proportional hazards (CPH) model [4]. However, a review of published cancer prognostic studies showed that missing covariate data are relatively common in clinical datasets and pose a great challenge to regression-based models [5]. Regression-based predictive models do not allow the input of incomplete predictors. In general, imputation of missing values should be performed before applying the developed model to new patients with missing predictors.

There are multiple methods for handling incomplete covariate data, including simple imputation, regression imputation, and multiple imputation (MI) [6] methods [7]. Simple imputation methods are commonly used to handle missing data, where the missing values are replaced by summary statistics such as mean, median, or mode. However, these methods tend to underestimate the variance of estimates and overlook correlations among variables, which can lead to biased inferences [8]. Regression imputation methods incorporate the possible association between missing values and other variables to generate more rational values. Nonetheless, these methods amplify the correlation among variables while underestimating the data variability [9]. The single imputation method does not consider the uncertainty related to missing values. MI can generate multiple sets of imputed values through different models and combine them into a final imputation. This approach ensures that the imputed dataset better matches the original data characteristics, thereby improving prediction accuracy of statistical models built on the imputed dataset. However, the computational complexity of MI poses challenges when dealing with large-scale datasets. Additionally, MI assumes that missing values occur randomly; if there is a specific missing pattern, such as a non-random mechanism, MI may lead to inaccurate conclusions. Owing to the limitations described above, these widely used imputation methods cannot achieve sufficient accuracy for datasets with missing values. Currently, there is no consensus on the most optimal approach for imputing missing data.

To address the challenge of missing data in clinical risk assessment, we applied a novel model titled conditional survival Bayesian networks (CSBN) [10], which combines the Bayesian network (BN) with the CPH model. Bayesian Networks handle missing data effectively by constructing a complex network structure of different factors, thus eliminating the need for imputing missing values before analysis. Given the evidence, the BN can infer the posterior probability distribution of query variables. This ability to update posterior probabilities makes it possible for Bayesian networks to solve the prediction problem even in the presence of missing data. As a result, BN has become a widely used approach for predicting the occurrence and progression of diseases as well as evaluating the effectiveness of different treatment options. For example, in cancer treatment, doctors could use BNs to predict patients' survival status and evaluate different treatment schemes, which can guide the development of optimal treatment plans. Moreover, BN's ability to support reasoning under uncertainty [11] has made it an extensively utilized tool in clinical diagnosis and risk prediction [12].

The remainder of this manuscript is organized as follows. In Section "Preliminaries", we review the basic concepts of the CPH model and the BN model, followed by the CSBN model. In Section "Data", we provide a description of the data used in this study. In Section "Methods", we designed a simulation study and provided a comprehensive description of the development of a prognostic predictive model for lung cancer. We evaluate the performance of the model and compare it with other imputation methods on the simulated datasets in Section "Results". In Section "Discussion", we discuss the advantages and limitations of our model. The conclusion is presented in Section "Conclusions". Finally, in Section "Future work", we consider future research in developing survival prediction models.

## Preliminaries

### Notations

In this section, we formalize the problem of survival analysis and describe how we combined the BN with the CPH model. The notations used in this paper are described in Table 1.

**Table 1:** Notations used in this manuscript

**Notation**	**Description**
$n$	number of features
$N$	number of samples
$p$	number of predictors in CPH model
${{\varvec{X}}}_{{\varvec{i}}}$	$1\times p$ vector of features for patient $i$ in CPH model
${{\varvec{X}}}_{{\varvec{B}}{\varvec{N}}}$	$1\times n$ vector of variables in Bayesian network model
$T$	observed time
$E$	indicator of event status
${x}_{i}$	the $i$-th variable for each patient
$h(t)$	the hazard function
${h}_{0}(t)$	the baseline hazard function
${H}_{0}(t)$	baseline cumulative hazard function
$S(t)$	survival probability function
${S}_{0}(t)$	baseline survival function

### Cox proportional hazards model

The CPH model [13] is a commonly used statistical regression model that examines the relationship between covariates and time-to-event outcomes. It combines the non-parametric baseline hazard with the parametric relative risk. In survival analysis, the primary objective is to estimate the survival probability function$S\left(t\right)=P(T>t)$ for each subject. This function provides the probability of a subject's survival time $T$ being beyond a given time $t$ [14].

Assuming we have data $[{\varvec{X}},T,E]$ for each patient, where ${\varvec{X}}=\{{x}_{1},{x}_{2},...{x}_{p}\}$ represents a p-dimensional vector comprising predictor variables. The indicator variable $E$ is used to denote the event status. Specifically, $E=1$ indicates that an event has occurred, while $E=0$ indicates that the event is absent (censored) during the follow-up period. The variable $T$ represents the time at which the event occurred (when $E=1$) or the censoring time (when $E=0$).

The hazard function, defined in Eq. (1), describes the instantaneous incidence of the event of interest at a given time t.

1 $$\begin{array}{lll}h\left(t\right)&=\underset{\Delta t\rightarrow0}{\mathit{lim}}\frac{\mathit{Pr}\left(t<T\leq t+\Delta t\vert T\geq t\right)}{\Delta t}\\&=\underset{\Delta t\rightarrow0}{\mathit{lim}}\frac{F\left(t+\Delta t\right)-F\left(t\right)}{\Delta t\cdot S\left(t\right)}=\frac{f\left(t\right)}{S\left(t\right)},\end{array}$$

where $F(\mathrm{t})$ stands for a cumulative event probability function, which can be formulated as $F\left(\mathrm{t}\right)=1-S(\mathrm{t})$. Here, S(t) denotes the survival probability function. Additionally, $f(\mathrm{t})$ represents the probability density function of $T$, which can be calculated as$f\left(t\right)=\frac{d}{dt}F\left(t\right)=-\frac{d}{dt}S\left(t\right)$.

The CPH assumes that the hazard function has the form, as shown in Eq. (2):

2 $$h(t\vert\boldsymbol X)=h_0(t)\exp(\boldsymbol X\boldsymbol\beta\boldsymbol'),$$

where ${h}_{0}(t)$ represents the baseline hazard function, which represents the underlying risk rate at time t when all covariates are set to their reference values. ${\varvec{X}}$ stands for the vector of predictors, and ${\varvec{\beta}}$ is a vector of unknown regression coefficients.

The baseline hazard function is estimated non-parametrically using methods such as the Breslow estimator or the Nelson-Aalen estimator [15]. The regression parameters ${\varvec{\beta}}$ are estimated by maximizing a partial likelihood function. Supposing the set of samples consist of $N$ observations $\left({X}_{i},{T}_{i},{E}_{i}\right){\left.\right|}_{\mathrm{i}=\mathrm{1,2},\dots \mathrm{N}}$, the partial likelihood function is defined in Eq. (3):

3 $$L(\boldsymbol{\beta})=\prod_{i{\in}D}\frac{\exp ({\boldsymbol{X}}_{\boldsymbol{i}}\boldsymbol{\beta}^{\boldsymbol{\prime}})}{\sum\nolimits_{j{\in}R ({T}_{i})}\;\exp ({\boldsymbol{X}}_{\boldsymbol{j}}\boldsymbol{\beta}^{\boldsymbol{\prime}})}$$

In the equation above, $D$ stands for the set of uncensored samples, defined as $D=\left\{i|{E}_{i}=1\right\}$, where ${E}_{i}=1$ indicates an event occurrence. $\mathrm{R}\left(\mathrm{t}\right)=\left\{j|{T}_{j}\ge t\right\}$ refers to the risk set at time t, which includes individuals who have not experienced the event by time t.

Once the parameters ${\varvec{\beta}}$ and the baseline hazard function are derived, the conditional survival probability function $S(\mathrm{t})$ for a given predictor vector ${\varvec{X}}$ can be obtained, as shown in Eq. (4),

4 $$\begin{array}{lll}S({t}\vert\boldsymbol{X})&=\exp&\left[-\int_0^th\;(s\vert X)\;ds\right]\\&=\exp&\left[-\int_0^th_0\;(t)\;ds\cdot\;\exp\;(\boldsymbol X\boldsymbol\beta\boldsymbol')\right]\\&=\exp&\left(-H_0\;(t)\right)^{\exp\;(\boldsymbol X\boldsymbol\beta\boldsymbol')}=S_0{(t)}^{\exp(\boldsymbol X\boldsymbol\beta\boldsymbol')}\end{array}$$

In this equation, ${H}_{0}(t)$ represents the baseline cumulative hazard function at time $t$, which is given by ${H}_{0}(t)={\int }_{0}^{t}{h}_{0}(t)dt$.

### LASSO for Cox proportional hazards model

The Lasso Cox model is a statistical technique that combines the Cox proportional hazards model with ${L}_{1}$regularization to achieve variable selection and parameter estimation [16]. The integration of ${L}_{1}$ regularization constrains certain parameters to 0, which allows for variable selection, reduces the complexity of the model, and results in greater interpretability while maintaining model validity.

The objective function of the Lasso Cox model consists of two main components: a log-partial likelihood term and an ${L}_{1}$ regularization term. The log-partial likelihood function, along with the penalty term for parameter estimation in the Cox model, is expressed as follows:

5 $$\sum_{i=1}^n\delta_i\left\{{\boldsymbol X}_i\boldsymbol\;\boldsymbol\beta\boldsymbol'\boldsymbol\;-\log\left[\sum_{j=1}^nI(T_j\geq T_i)\;\exp\;({\boldsymbol X}_{i\boldsymbol\;}\boldsymbol\beta\boldsymbol')\right]\right\}\;-\lambda\;\sum_{k=1}^p\vert\beta_k\vert,$$

Where $n$ is the number of the samples, $p$ is the dimensionality of ${\varvec{\beta}}$, $\uplambda$ is a regularization parameter, and $\sum_{k=1}^{p}\left|{\beta }_{k}\right|$ is the ${L}_{1}$ norm. The optimal value of $\uplambda$ is selected using cross-validation to strike a balance between bias and variance in the model.

The log-partial likelihood term assesses the goodness-of-fit of the model to the data, while the ${L}_{1}$ regularization term controls the complexity of the model.

The Lasso Cox model effectively eliminates less significant variables from the model, leading to improved generalization ability and model stability, while simultaneously mitigating overfitting issues.

### BN

A BN is a directed acyclic graph (DAG) that represents probabilistic dependencies among a set of variables. Each node in the graph corresponds to a random variable, and a directed edge between two nodes indicates the probabilistic relationship between the variables in the network [17].

Let ${{\varvec{X}}}_{{\varvec{B}}{\varvec{N}}}=\{{x}_{1},{x}_{2},...{x}_{n}\}$ be a set of $n$ variables. A BN over ${{\varvec{X}}}_{{\varvec{B}}{\varvec{N}}}$ is denoted as a pair $B(\mathrm{G},\Theta )$, where $\mathrm{G}$ represents the structure of the DAG and $\Theta$ represents the joint probability distribution of the DAG [18]. Specifically, if there is an edge from node ${x}_{i}$ to node ${x}_{j}$, ${x}_{i}$ is referred to as the parent of ${x}_{j}$, and ${x}_{j}$ is the child of ${x}_{i}$. The parents of ${x}_{i}$ in the network are denoted as ${\Pi }_{{x}_{i}}$. Under the assumption of the BN [19], the joint probability of the global distribution can be decomposed into a product form as given in Eq. (6):

6 $$\mathrm\Theta\;=\;P\;(x_1,x_1,..,x_n)\;=\prod_{i=1}^nP\left\{x_1\vert{\textstyle\prod_{x_1}}\right\}$$

where $P\left\{{x}_{i}|{\Pi }_{{x}_{i}}\right\}$ represents the local conditional probability distribution of ${x}_{i}$.

The BN model is trained in two steps: structure learning and parameter learning [20]. In structure learning, the goal is to identify an appropriate DAG that represents the relationships among the nodes. Parameter learning, on the other hand, aims to determine the conditional probability distribution of each node given its parents.

For discrete variables, each node in the BN is associated with a conditional probability table (CPT). The CPT contains the probabilities of each possible value of the node, given all possible combinations of states of its parents. The CPT provides the conditional probabilities necessary for inference and prediction in the BN model.

### Conditional Survival BNs

Conditional Survival Bayesian Network (CSBN) is a special type of BN denoted as $B({\mathrm{G}}^{\mathrm{^{\prime}}},{\Theta }^{\mathrm{^{\prime}}})$, where ${\mathrm{G}}^{\mathrm{^{\prime}}}$ consists of a set of discrete nodes $(D$) and a survival node $(E)$. The process of learning CSBN involves two steps: learning a BN model and combining it with the Cox Proportional Hazards (CPH) model.

In the first step, the general BN for the discrete node $D$ is learned using standard BN learning techniques. This step involves applying standard BN learning algorithms to identify the probabilistic dependencies among the discrete variables.

In the second step, the CPH model is utilized to extract the most significant risk factors associated with survival. These risk factors are then integrated into the previously obtained BN by connecting them to the survival node $E$. This integration is done by manually adding directed edges from the predictors of the CPH model to the survival node E. The parent set of $E$, denoted as ${\Pi }_{\mathrm{E}}={\varvec{X}}=\{{x}_{1},{x}_{2},...{x}_{p}\}$.

By combining the BN model with the CPH model, the structure of the CSBN is obtained, which includes the previously learned BN structure along with the additional edges connecting the predictors of the CPH model to the survival node $E$.

To determine the values for the CPT of the survival node, we estimate ${P}_{E|X}\left(E|{\varvec{X}},T\right)$, which represents the conditional probability of the event occurring before time $T$ given the states of the parents ${\varvec{X}}$. This estimation is done using the CPH model and the following general formula, as shown in Eq. (7).

7 $$\begin{array}{lll}P_{E\vert X}\;(E=1\vert\boldsymbol X,T=t)\\=P(E=1\vert{\textstyle\prod_E},T=t)\\=1-S_0{\;(t)}^{\exp({\textstyle\sum_{i=1}^p}\beta_ix_i)},\end{array}$$

where ${\mathrm{S}}_{0}(\mathrm{t})$ is the baseline survival probability at time $t$, which is calculated in the CPH model. The variable ${x}_{i}$ represents the value of the $i$-th covariate (assume baseline value standardized to 0), ${\beta }_{i}$ represents the estimated regression coefficient corresponding to the $i$-th covariate ${x}_{i}$, and $p$ stands for the number of covariates in the CPH model.

## Data

The study utilized a dataset comprising 5,240 lung cancer patients who received treatment at the Weihai Municipal Hospital from May 2013 to May 2021. The inclusion criteria involved patients diagnosed with primary lung cancer within the specified timeframe, while those with abnormal ID formats were excluded from the analysis. The primary endpoint of the study was overall survival (OS), defined as the time from the initial lung cancer diagnosis to the last follow-up or death. The outcomes were obtained from the death registry database maintained by the Shandong Provincial Center for Disease Control and Prevention.

Among the initial cohort of 5,240 participants in this study, 51.9% were male and 48.1% were female. The median age of the participants was 63 years, ranging from 22 to 92 years. The median follow-up period was 2.04 years, with an interquartile range of 347 to 1,294 days. During the follow-up, 21% of the patients were identified as deceased.

This study incorporated a total of 26 variables, including demographic characteristics, comorbidities, laboratory and clinical features, as well as diagnostic variables. The dataset consisted of both continuous and discrete variables. To apply the widely used discrete Bayesian Network (BN) method, the continuous variables were discretized. For instance, the variable of age was discretized into four groups: < 45, 45–59, 60–74, and > 74 years old. Laboratory characteristics were categorized via tertile division, with T_1_, T_2_, and T_3_ representing the first, second, and third tertiles of the dataset, respectively.

## Methods

### Simulation study

To assess the reliability of the CSBN model, a simulation experiment was conducted using datasets with varying missing rates. Simulated data was employed to evaluate the predictive performance of the CSBN model.

In simulated experiments of this study, covariate characteristics were the same distribution as the Weihai lung cancer cohort. The event time in the simulations was modeled using a log-normal distribution, with the parameter determined by XW', where W' was obtained through log-normal regression in the original data and X represented the feature vector. The censoring time was modeled using a Weibull distribution, with parameters estimated from regression analysis on the original data.

The simulation procedure was repeated 500 times to generate a synthetic dataset of 6,000 patients for each missing-rate scenario. The simulated datasets were divided into training and test sets in a 1:1 ratio. To introduce missing values, we randomly removed observations from the test data, with the proportion of missing values ranging from 10% to 40% in increments of 10%.

We compared the CSBN model with three other commonly used imputation methods, namely KNN, MICE, and missForest. The recently proposed missForest method uses random forest to predict the missing values [21]. The KNN algorithm [22] is based on the nearest-neighbor search, where each missing value is replaced by a weighted mean of k-nearest observation values. MICE [23] is a multiple imputation method that iteratively imputes missing values by fitting conditional models for each variable.

For this experiment, we fixed the number of nearest neighbors at five for the KNN algorithm, and the number of multiple imputations was set at 10 for MICE. The simulated datasets were imputed using these three imputation methods and then utilized in a multivariable Cox model to make survival predictions.

We compared the CSBN model with three imputation methods on simulated datasets that had varying proportions of missing values. The accuracy of different predictive models was compared using their average AUC values across 500 simulated datasets. The simulation aimed to evaluate the impact of different imputation methods on the predictive performance of lung cancer prediction models.

### Statistical analysis

**Fig 1:**
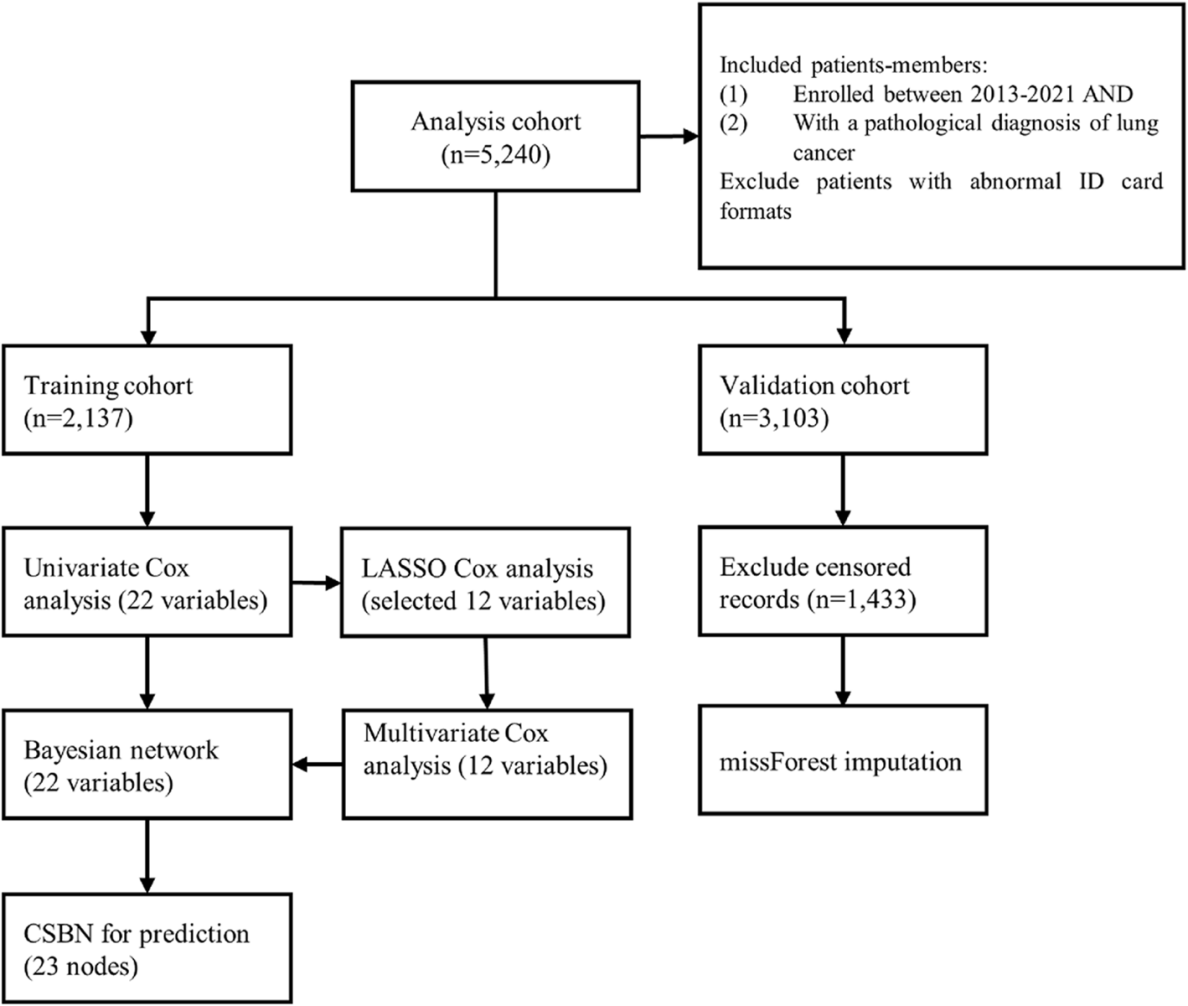
Flowchart of prediction model development process

We searched for potential predictors of lung cancer by conducting a univariable analysis with Cox proportional hazards regression within the derivation cohort. We conducted a LASSO penalty with tenfold cross-validation to select prognostic predictors of lung cancer. The predictive ability of our models was assessed using the area under the receiver operating characteristic curve (AUC), Harrell concordance index (C-index), and calibration curves [24, 25]. Furthermore, to evaluate their clinical utility, decision curve**a**nalyses (DCA) were carried out [26]. We employed R software version 4.0.5 to build the model and perform statistical analyses with the following packages: survival, bnlearn, pROC, coxph. The flow chart of the prediction model development process is shown in Fig. 1.

### Variable selection

The data was divided into training and validation cohorts based on the completeness of patient's information. The training cohort comprised 2,137 participants with no missing covariates, while the validation cohort had 3,103 participants with missing values. The baseline characteristics of both cohorts were stratified by survival outcome and summarized in Supplementary Table 1.

The training cohort was utilized for developing the predictive model. To construct a predictive model and identify potential factors that may be associated with the risk of death in lung cancer patients, all candidate factors were screened by univariate Cox regression analysis with a significance threshold of $p<0.05$. The complete form of univariate Cox regression analyses for overall survival is presented in Table 2. The univariate Cox regression analyses revealed that smoking, older age, pleural effusion, worse pathological stage, lung abscess, pulmonary heart disease, interstitial lung disease (ILD), pulmonary embolism, respiratory failure, higher red blood cell count, higher fibrinogen, higher eosinophil, and being male were associated with a higher mortality risk from lung cancer.

**Table 2:** Univariate Cox regression analysis and multivariate Cox regression analysis

	**Univariate analysis**			**Multivariate analysis**		
**Characteristic**	**Beta**	**HR (95% CI)**	***P***	**Beta**	**HR (95% CI)**	***P***
Gender						
Female	Reference			Reference		
Male	0.799	2.224 (1.864–2.654)	<0.001	0.074	1.077 (0.839–1.383)	0.560
Age						
(18,44]	Reference			Reference		
(44,59]	0.784	2.190 (1.117–4.296)	0.023	−0.037	0.964 (0.487–1.909)	0.916
(59,74]	1.107	3.025 (1.560–5.865)	0.001	0.108	1.114 (0.567–2.191)	0.754
(74,92]	1.758	5.800 (2.897–11.612)	<0.001	0.509	1.664 (0.822–3.372)	0.157
Smoking						
Never	Reference			Reference		
Current	1.033	2.809 (2.321–3.398)	<0.001	0.585	1.795 (1.306–2.466)	<0.001
Former	0.628	1.874 (1.497–2.345)	<0.001	0.186	1.205 (0.881–1.647)	0.243
Drinking						
no	Reference			Reference		
yes	0.686	1.986 (1.672–2.359)	<0.001	−0.172	0.842 (0.650–1.092)	0.195
NSCLC						
no	Reference			Reference		
yes	−1.117	0.327 (0.265–0.404)	<0.001	−0.09	0.914 (0.736–1.135)	0.416
Radiotherapy	0.431	1.539 (1.290–1.836)	<0.001			
Chemotherapy	0.555	1.743 (1.475–2.060)	<0.001			
Targeted therapy	0.656	1.928 (1.627–2.284)	<0.001	−0.459	0.632 (0.525–0.761)	<0.001
COPD	0.633	1.884 (1.591–2.230)	<0.001	0.195	1.216 (1.009–1.465)	0.040
Pneumonia	0.93	2.535 (2.145–2.996)	<0.001	0.125	1.133 (0.946–1.359)	0.176
Pleural effusion	1.063	2.894 (2.431–3.445)	<0.001			
STAGE						
I	Reference			Reference		
II	1.888	6.607 (3.105–14.058)	<0.001	1.778	5.916 (2.771–12.631)	<0.001
III	3.234	25.393 (14.608–44.140)	<0.001	3.027	20.644 (11.716–36.377)	<0.001
IV	3.897	49.263 (28.908–83.952)	<0.001	3.716	41.091 (23.662–71.356)	<0.001
URI	−0.445	0.641 (0.443–0.926)	0.018			
Lung abscess	0.876	2.402 (1.322–4.365)	0.004			
Pulmonary embolism	1.138	3.121 (2.037–4.783)	<0.001			
Pulmonary heart disease	1.013	2.753 (1.954–3.877)	<0.001			
ILD	1.022	2.779 (1.980–3.899)	<0.001	0.416	1.515 (1.063–2.159)	0.021
Respiratory failure	1.368	3.926 (2.902–5.313)	<0.001	0.628	1.873 (1.362–2.577)	<0.001
Red blood cell count						
T_1_	Reference					
T_2_	0.517	1.678 (1.300–2.166)	<0.001			
T_3_	1.039	2.825 (2.219–3.598)	<0.001			
Eosinophil						
T_1_	Reference					
T_2_	0.517	1.678 (1.300–2.166)	<0.001			
T_3_	1.039	2.825 (2.219–3.598)	<0.001			
Fibrinogen						
T_1_	Reference			Reference		
T_2_	0.804	2.234 (1.711–2.917)	<0.001	0.276	1.318 (1.004–1.730)	0.047
T_3_	1.754	5.780 (4.537–7.363)	<0.001	0.57	1.769 (1.374–2.277)	<0.001
Direct bilirubin						
T_1_	Reference					
T_2_	−0.396	0.673 (0.556–0.816)	<0.001			
T_3_	−0.774	0.461 (0.370–0.575)	<0.001			

**Fig 2:**
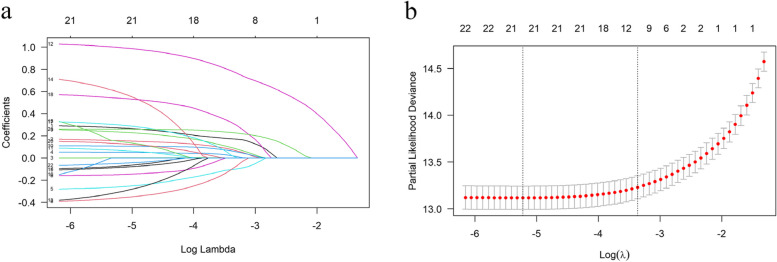
Feature selection using the LASSO Cox regression model. **a** LASSO coefficient profiles of the 22 predictors in the training cohort. **b** Cross-validation to select the optimal regularize parameter $\uplambda$ based on the error within one standard error range of the minimum, 12 predictors selected using LASSO Cox regression analysis

To further simplify the model and alleviate the problem of overfitting, LASSO regression was introduced for feature selection. The LASSO method is a useful feature selection method that reduces the coefficient of insignificant variables to 0 with a penalty function. Twenty-two variables initially screened through univariate Cox regression analyses were considered as potential variables for the LASSO Cox model. The optimal penalization coefficient for the model was selected through tenfold cross-validation. By utilizing the LASSO selection procedure in multivariate Cox proportional hazards models, twelve independent prognostic predictors for lung cancer were identified (Fig. 2). These predictors were then integrated into the Bayesian network (BN) by directly linking them to the lung cancer survival outcome node.

The final multivariable Cox regression model was constructed using all the prognostic predictors obtained from the LASSO selection procedure. The regression coefficients and hazard ratios of these prognostic factors are presented in Table 2. In the multivariate analysis, several factors were found to be independent prognostic factors for poorer overall survival in lung cancer patients, which aligns with previous reports [27–29]. These factors include worse pathological stage, smoking, older age, COPD, higher fibrinogen level, and pneumonia. We also found that alcohol drinking had a protective effect on lung cancer, but this was not statistically significant ($P=0.195$). Previous studies have suggested that low or moderate alcohol consumption is associated with a reduced risk of lung cancer death [30].

### The process of generating the BN model

The BN model was constructed using the 22 significant variables identified through univariable analysis with Cox proportional hazards regression. The network structure was determined using a data-driven approach that combined a tabu-search algorithm [31] with prior knowledge from the medical literature. For instance, based on available medical evidence, the nodes representing age and gender were allowed to have a direct influence on smoking, while no variable was permitted to influence age and sex [32]. Moreover, considering that smoking is the primary cause of COPD, COPD was represented as a child node of smoking in the BN [33].

**Fig 3:**
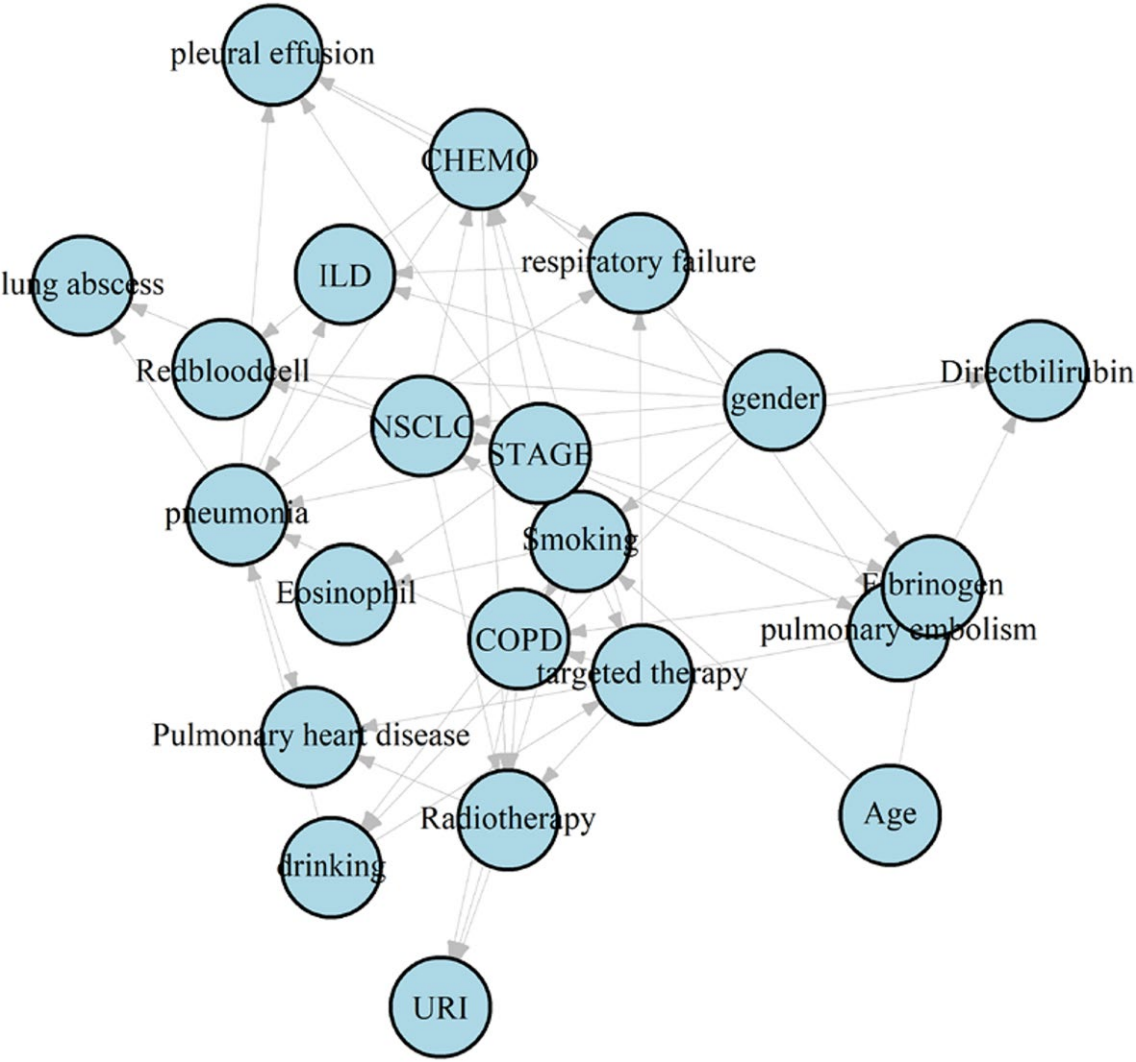
Bayesian network structure

To achieve a high-quality and robust network structure, this study employed Bootstrap and model averaging methods in the network structure learning process [34]. These methods were used to obtain high-confidence connections as prior information for network construction. The high-confidence connections were treated as a whitelist within the Bayesian network. Parameter learning was carried out using the maximum likelihood estimation algorithm to derive the conditional probability tables for each node. The visualization of the network topology is depicted in Fig. 3.

### Combination of the BN and Cox model

In order to leverage the strong estimation capabilities of Cox regression models for survival data along with the effective inference capabilities of BN, an additional node representing the survival outcome of lung cancer patients was incorporated into the constructed BN. This survival outcome node provided information on whether each patient experienced mortality (valued 1) or survival (valued 0). The independent prognostic factors identified through LASSO Cox regression analysis were directly linked to the lung cancer survival outcome node. The conditional probability table for the survival outcome node was determined using Eq. (7).

The final conditional survival BN model was obtained by re-estimating the parameters of the BN. The parameters of the survival outcome node were specifically determined using the Cox proportional hazards model. The results of the Cox model are embedded in a Bayesian network in the form of a conditional probability table. The likelihood weighting inference algorithm [35] in the Bayesian network was used to determine three-year survival probabilities in lung cancer patients.

## Results

### Simulation experiment for varying missing rates

**Fig 4:**
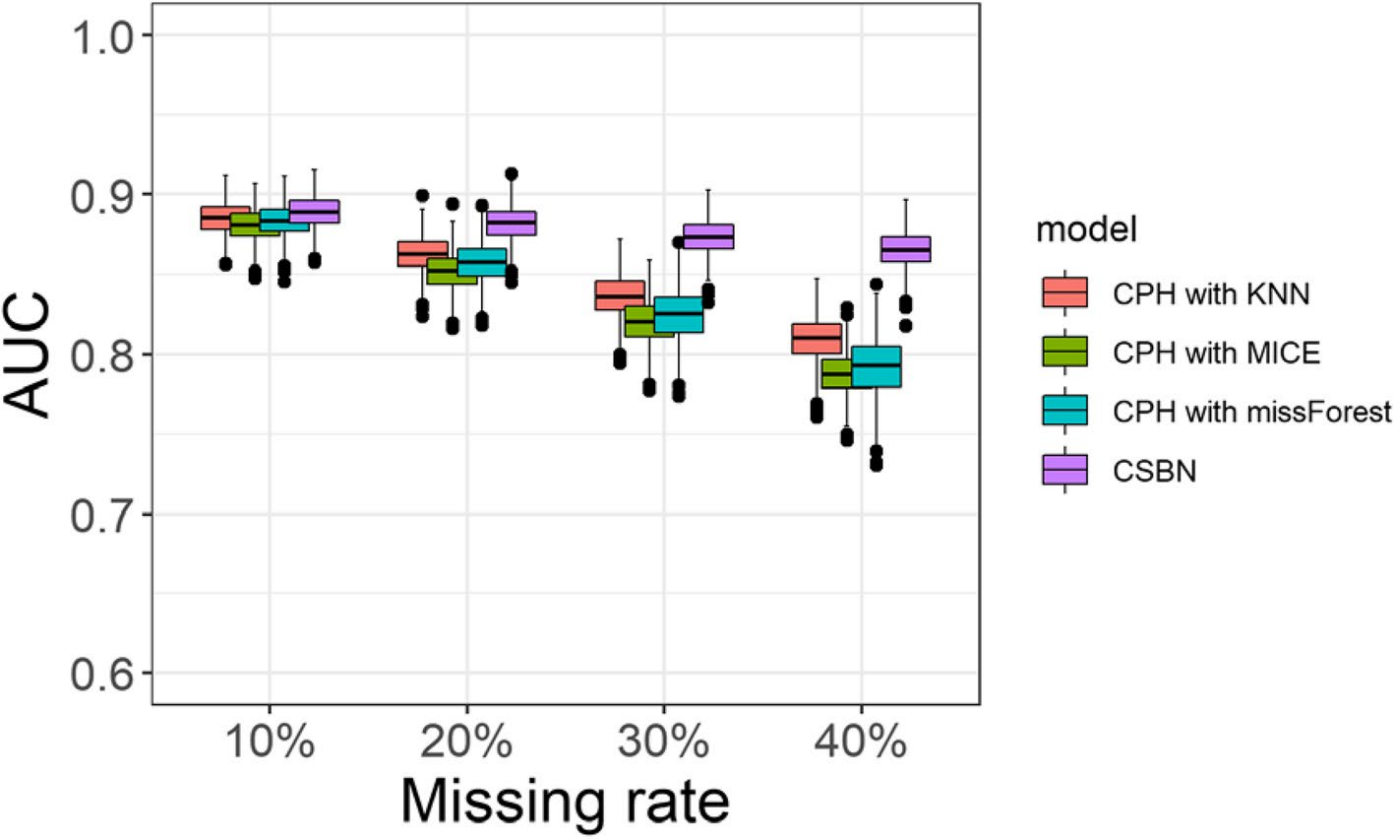
Boxplots of the AUCs for models with different imputation methods on the following four simulated datasets. 1. Proportion of missing values = 10%, 2. Proportion of missing values = 20%, 3. Proportion of missing values = 30%, 4. Proportion of missing values = 40%. Note that the CSBN model attained the best performance. As the missing rate increased, the average AUCs of the Cox model decreased significantly, but the average AUCs of the CSBN were both good and stable

The results depicted in Fig. 4 display the AUCs of various survival prediction models on simulated datasets with different proportions of missing values. As expected, the AUC decreased with an increase in the proportion of missing values in the validation dataset. At each missing proportion level, the CSBN model demonstrated the highest performance, followed by KNN. Moreover, the performance gap between CSBN and KNN widened as the proportion of missing values increased. The mean performance of missForest and MICE was nearly identical. Additionally, we observed that the AUC of the CSBN model remained relatively stable compared to the other three imputation methods as the missing rate escalated from 10% to 40%.

When evaluating the impact of missing values, we found that the CSBN model exhibited good performance even when the missing rate reached 40%, maintaining a strong discriminatory ability with an AUC higher than 0.8. In contrast, the AUC of the CPH model experienced a more significant decline when utilizing general imputation methods. This result provides evidence that the CSBN model surpasses commonly used imputation methods and exhibits superior robustness in handling missing data.

### Validation cohort performance

Having demonstrated the superior performance of the CSBN model on simulated datasets, we next verify whether it can maintain this level of performance in real-world cases. To verify the validity of the CSBN model, the remaining 3,103 medical records in the validation cohort were used as test samples. In this cohort, we excluded patients who did not complete the three-year follow-up period, resulting in 1,433 samples used for internal model validation.

We evaluated the predictive performance of the CSBN model in terms of discrimination and calibration. Discrimination was assessed using the concordance statistic and the AUC. The calibration plot was employed to assess the agreement between the predicted and observed risk of lung cancer.

**Fig 5:**
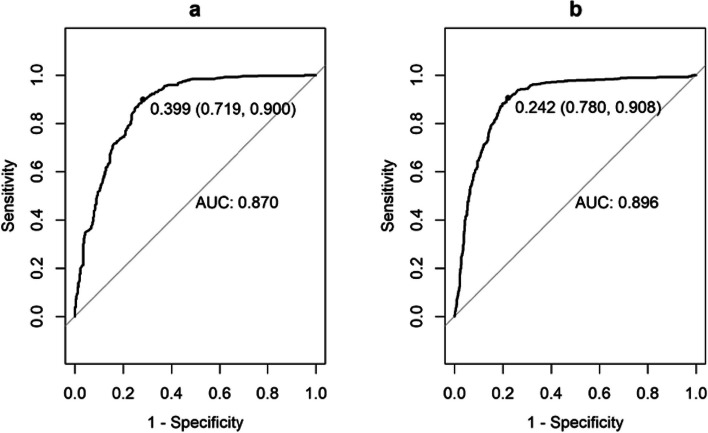
ROC curves of the CSBN. The ROC curves of the CSBN predicting three-year overall survival in the training cohort (**a**) and validation cohort (**b**)

**Fig 6:**
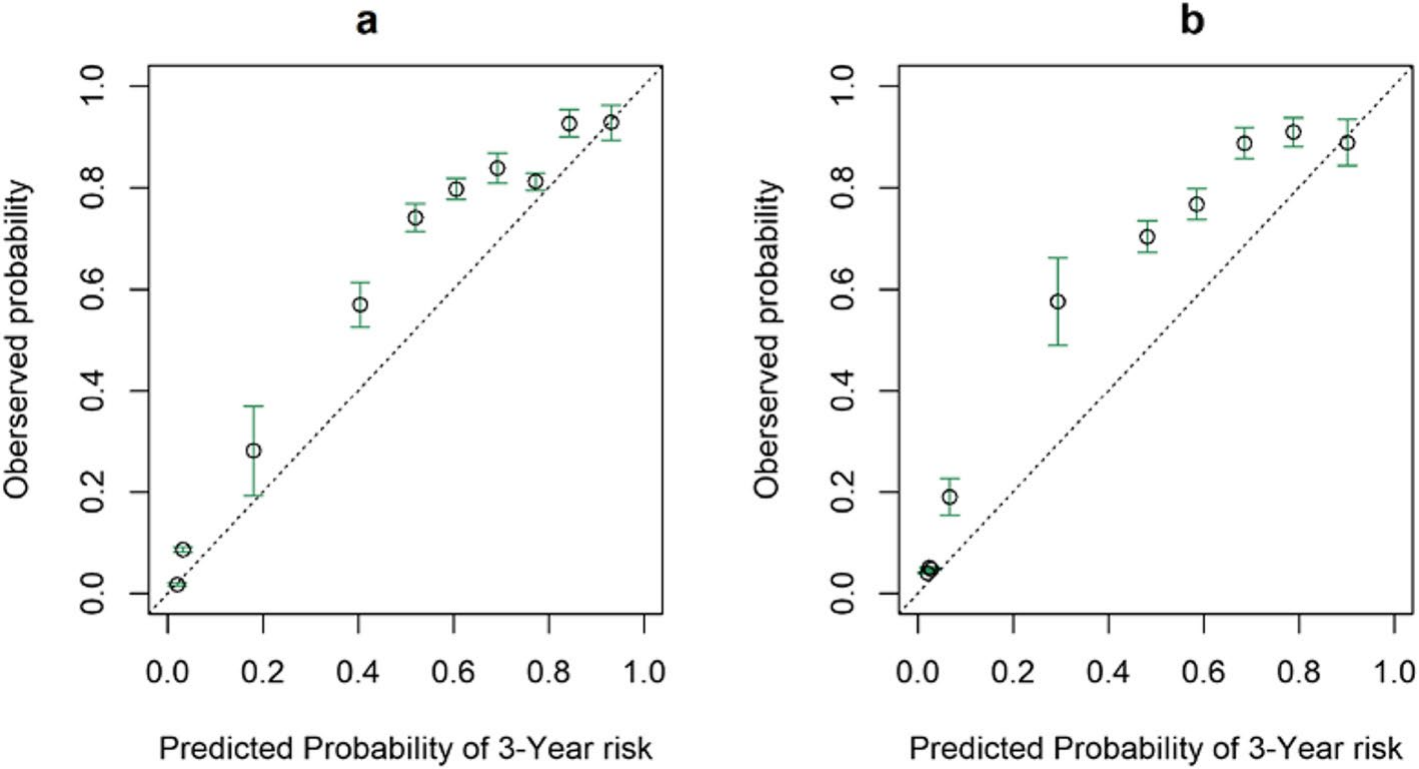
Calibration plots of the CSBN. The calibration curves of the CSBN for predicting three-year overall survival in the training cohort (**a**) and validation cohort (**b**)

The CSBN model exhibited good discrimination for three-year survival, with an AUC of 0.870 (95% CI: 0.845~0.895) in the training cohort and 0.896 (95% CI: 0.878~0.913) in the validation cohort (Fig. 5). The calibration plot was generated by plotting the observed and predicted risk in each decile of predicted risk. The calibration curve for three-year survival in the validation cohort demonstrated a high level of consistency between the predicted probabilities and the observed probabilities (Fig. 6).

To further evaluate the performance, we compared the CSBN model with the CPH model. We assessed the predictive accuracy of the CPH model in the validation cohort by imputing the incomplete test samples using the non-parametric missing data imputation method implemented via the R-package of missForest. The imputed data was then used to make three-year survival predictions using the CPH model.

The CPH model alone yielded an AUC of 0.863 (95% CI, 0.848~0.877) for three-year overall survival in the validation cohort. In contrast, the CSBN model achieved a slight improvement in predictive power in the presence of missing predictors.

To compare the clinical utility between our prediction model and the CPH model, we conducted decision curve analysis. DCA evaluates the clinical usefulness by calculating the net benefit, which involves a trade-off between true positive rates and false-positive rates. Specifically, we calculated the net benefit at a range of risk thresholds for each model.

**Fig 7:**
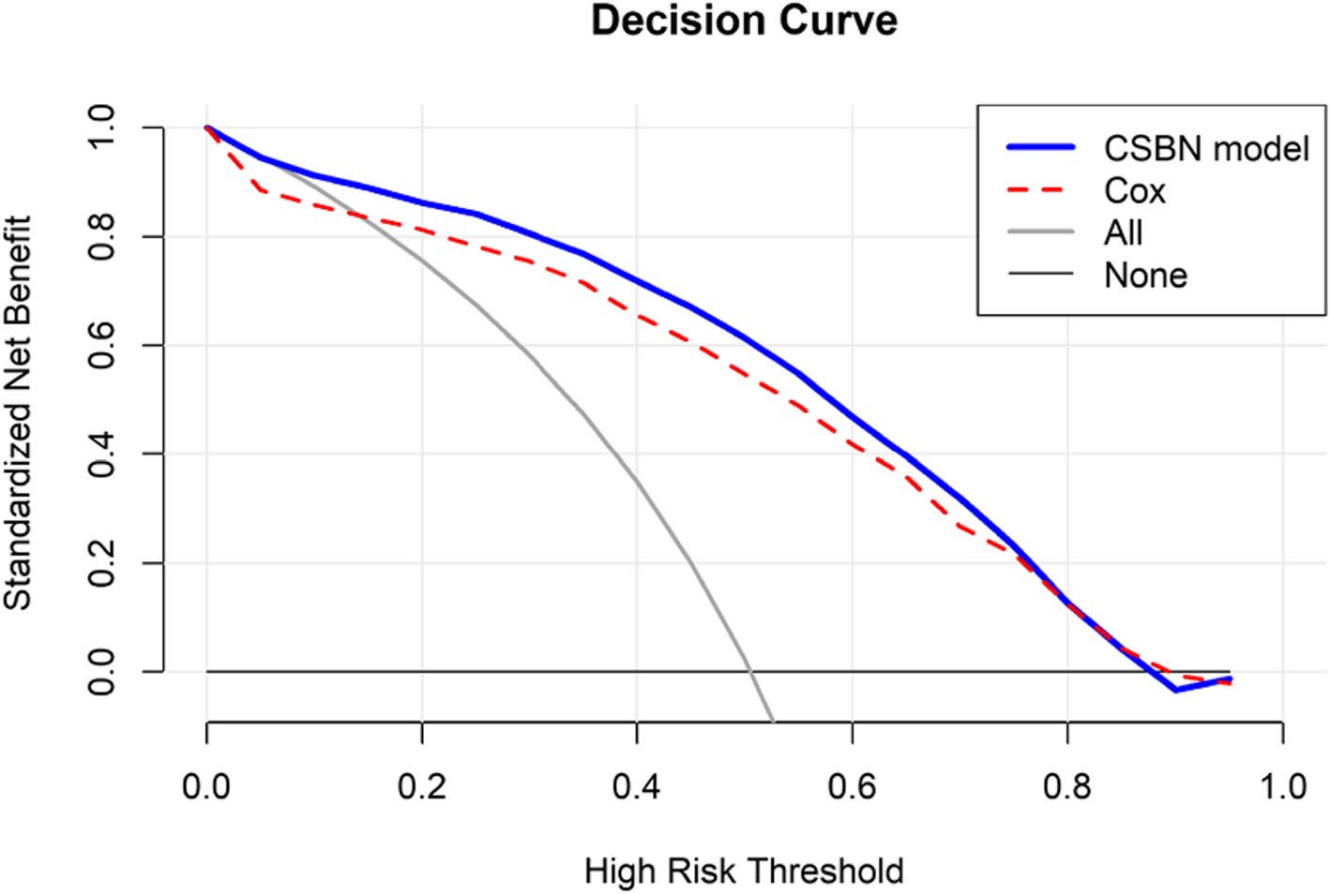
Decision curve analysis for the comparison of the net benefit between the CSBN model (blue line) with the CPH model (red dotted line). The CSBN model achieved a higher net benefit compared with the CPH model

As shown in Fig. 7, the standardized net benefit of our model surpassed that of the CPH model within the range of threshold probabilities up to 70%. This demonstrates the utility contribution of the BN approach.

## Discussion

In this study, we utilized a joint model that combines the Cox proportional hazards model with the Bayesian network (BN) to predict three-year survival in lung cancer patients. The established prognostic model for lung cancer was evaluated using two performance metrics, namely the AUC and calibration, and internal validation demonstrated high discrimination and calibration.

Through simulation studies, we discovered that the BN strategy significantly enhances discrimination compared to missForest, KNN, and MICE methods when dealing with high ratios of missing data. Our findings provide support for the use of the CSBN model as an effective tool for risk prediction, particularly when clinical records of patients are incomplete.

The CSBN model utilizes Bayesian networks to reason about the probability of event occurrence based on the available variables and their conditional probability relationships, effectively addressing the challenge of risk assessment with incomplete information. The proposed approach in this study has a fourfold contribution. (1) The proposed prediction model compensated for the shortcomings of the CPH model, where predictions should be made based on all known variables in the model. This was achieved by inference methodologies of the BNs model. (2) BNs have the ability to incorporate expert knowledge and observational data to identify the conditional independence between risk factors. They also provide a visual tool that intuitively reflects the relationship between survival and prognostic factors. (3) Since the model is based on readily available covariates in daily clinical practice, it can serve as a prognostic instrument for individual lung cancer patients, assisting clinicians in decision-making processes. (4) As BNs derive predictions through a probabilistic framework, the results can be explained from a probability perspective.

The CSBN model effectively addresses the challenges of missing data in risk assessment while maintaining high prediction accuracy. However, we must acknowledge the limitation that the CPT of the survival outcome node grows exponentially as the number of its immediate parent nodes increases [36]. To avoid the problem of dimensionality and expand the applicability of the CSBN model, we employed variable selection techniques to reduce the complexity of the CPH model. There are many existing variable selection techniques such as optimal subset variable selection, stepwise regression, and LASSO [37]. In this study, the widely used lasso penalty was utilized for variable selection.

## Conclusions

In this study, we used a hybrid solution that combined a CPH model and a BN model to solve the problem of missing data in prognostic research for lung cancer. Internal validation suggests that our model has good predictive performance in both discrimination and calibration. In addition, the simulation results show that the BN imputation methods are more efficient than other widely used imputation methods and relatively robust among various missing rates of the data. The BN model effectively handles missing data and enhances the robustness of the model through probabilistic inference.

Our findings suggest that the BN model has promising potential in improving the accuracy and reliability of survival prediction in the presence of missing data. These results provide valuable insights into the application of BN models in healthcare and medical research.

## Future work

Survival analysis plays a critical role in various fields, but the presence of missing data often poses challenges in accurately estimating survival probabilities and making reliable predictions. In this study, we developed and optimized a Bayesian network model for survival analysis. The BN model captures missing data variability from a probabilistic standpoint, resulting in improved model robustness.

Accurately assessing patient risk is crucial for making personalized treatment decisions in clinical practice. Future research can explore further optimization and improvement of the model. Introducing additional clinical features and biomarkers provides a potential avenue to enhance the accuracy of the models. By incorporating a broader range of variables, we can improve the model's predictive power and its applicability in real-world clinical settings. Ultimately, these advancements in personalized medicine can lead to improved patient outcomes and more effective healthcare services.

### Supplementary Information

**Additional file 1:** **Table 1.** Baseline characteristics of the training cohort and validation cohort stratified by survival outcome.

## Data Availability

The datasets are available from the corresponding author upon reasonable request.

## Abbreviations

AUC: Area under the receiver operating characteristic curve
CI: Confidence interval
ROC: Receiver operating characteristic
BN: Bayesian Network
HR: Hazard ratio
CI: Confidence interval
CPT: Conditional probability table
CPH: Cox Proportional Hazards
DCA: Decision curve analyses
NSCLC: Non-small cell lung cancer
COPD: Chronic obstructive pulmonary diseases
URI: Upper respiratory tract infections
ILD: Interstitial Lung Disease
